# Hourly PWV Dataset Derived from GNSS Observations in China

**DOI:** 10.3390/s20010231

**Published:** 2019-12-31

**Authors:** Qingzhi Zhao, Pengfei Yang, Wanqiang Yao, Yibin Yao

**Affiliations:** 1 College of Geomatics, Xi’an University of Science and Technology, Xi’an 710054, China; 19210061031@stu.xust.edu.cn (P.Y.); sxywq@xust.edu.cn (W.Y.); 2 School of Geodesy and Geomatics, Wuhan University, Wuhan 430000, China; ybyao@whu.edu.cn

**Keywords:** GNSS, ERA5, PWV, diurnal variation

## Abstract

The rapid variation of atmospheric water vapor is important for a regional hydrologic cycle and climate change. However, it is rarely investigated in China, due to the lack of a precipitable water vapor (PWV) dataset with high temporal resolution. Therefore, this study focuses on the generation of an hourly PWV dataset using Global Navigation Satellite System (GNSS) observations derived from the Crustal Movement Observation Network of China. The zenith total delay parameters estimated by GAMIT/GLOBK software are used and validated with an average root mean square (RMS) error of 4–5 mm. The pressure (P) and temperature (T) parameters used to calculate the zenith hydrostatic delay (ZHD) and weighted average temperature of atmospheric water vapor (*T_m_*) are derived from the fifth-generation reanalysis dataset of the European Centre for Medium-Range Weather Forecasting (ECMWF ERA5) products. The values of P and T at the GNSS stations are obtained by interpolation in the horizontal and vertical directions using empirical formulas. *T_m_* is calculated at the GNSS stations using the improved global pressure and temperature 2 wet (IGPT2w) model in China with an RMS of 3.32 K. The interpolated P and T are validated by interpolating the grid-based ERA5 data into radiosonde stations. The average RMS and bias of P and T in China are 2.71/−1.11 hPa and 1.88/−0.51 K, respectively. Therefore, the error in PWV with a theoretical RMS of 1.85 mm over the period of 2011–2017 in China can be obtained. Finally, the hourly PWV dataset in China is generated and the practical accuracy of the generated PWV dataset is validated using the corresponding AERONET and radiosonde data at specific stations. Numerical results reveal that the average RMS values of the PWV dataset in the four geographical regions of China are less than 3 mm. A case analysis of the PWV diurnal variations as a response to the EI Niño event of 2015–2016 is performed. Results indicate the capability of the hourly PWV dataset of monitoring the rapid water vapor changes in China.

## 1. Introduction

Atmospheric water vapor is a key component of the troposphere, and it affects the global/regional water cycles and climate change [1]. Water vapor is also an important greenhouse gas, which is highly related to the heat feedback function. Therefore, knowledge of rapid water vapor variations is important for analyzing global/regional water vapor distributions [2]. However, the distribution of atmospheric water vapor with high temporal–spatial resolutions is still difficult to monitor, due to the shortcomings of traditional techniques for water vapor monitoring. Precipitable water vapor (PWV), which is used to reflect the content of atmospheric water vapor, is the value corresponding to the conversion of all atmospheric water vapor into liquid water in the air column of a unit area from the surface to the troposphere high. A number of techniques have been developed to monitor PWV, and they include radio sounding, water vapor radiometer (WVR), solar photometer, and remote sensing. The use of a sounding balloon is the traditional method for obtaining PWV; the balloon is only launched twice daily, due to its high cost [3,4]. Therefore, radiosonde data with high temporal–spatial resolutions cannot be guaranteed, due to its disadvantages. Another technique to obtain PWV is WVR, which has many advantages, such as high time resolution, convenient processing, and resistance against the influence of climate [5]. However, this method remains limited because of its disadvantages, which include high cost, huge size, and fixed location [6]. Proposed by Bevis et al. (1992), GNSS is an alternative technique to obtain PWV, and its advantages include its capability to perform under all weather conditions and with low cost and high temporal–spatial resolutions [7].

As mentioned previously, PWV can be obtained using GNSS observations in the following way: when the satellite signal passes through the troposphere, it will produce refraction and bending, due to the influence of atmospheric effects. Therefore, a delay is generated, which is referred to as the zenith tropospheric delay (ZTD) [8]. ZTD consists of zenith hydrostatic delay (ZHD) and zenith wet delay (ZWD). The former accounts for about 90% of ZTD, and it can be accurately estimated using surface pressure (P) on the basis of an empirical model [9]. The latter can be calculated by extracting ZHD from ZTD. Temperature (T) can be used to obtain the weighted mean temperature of atmospheric water vapor (*T_m_*) and convert ZWD to PWV [7,10]. Unfortunately, most fixed GNSS stations are not equipped with meteorological sensors. Hence, the T and P used to convert ZTD to PWV are unavailable. To overcome the lack of meteorological parameters, Wang et al. (2007) and Jin et al. (2009) proposed a method for obtaining P by interpolating the corresponding meteorological data of synoptic stations near GNSS stations while deriving *T_m_* from the National Center for Environmental Prediction/National Center for Atmospheric Research [11,12]. Zhao et al. (2018a) used the P and *T_m_* of IGS stations derived from the Global Geodetic Observing System (GGOS) atmosphere to calculate PWV on a global scale [13,14]. Zhang et al. (2017) also used the interpolated P and T from the European Centre for Medium-Range Weather Forecasting (ECMWF) to obtain the PWV in China [15]. Zhao et al. (2019) used the layered products from the ECMWF ERA-Interim reanalysis data to calculate ZHD and *T_m_* in China [16].

Although some efforts have been made to obtain the PWV at GNSS stations, the temporal resolutions of reanalysis data or synoptic data are 6 and 3 h, respectively. These values indicate that a rapid change in atmospheric water vapor reflected by PWV may not be monitored. Therefore, obtaining GNSS-derived PWV with a relatively high temporal resolution (hourly) is a key issue that needs to be resolved. Fortunately, the ECMWF released its fifth-generation atmospheric reanalysis data of the global climate (ERA5) in 2018 with a temporal resolution of 1 h, which makes obtaining hourly GNSS-derived PWV possible. Therefore, the hourly P and T derived from ERA5 are used in the present study to calculate ZHD and *T_m_* on the basis of the Saastamoinen and Bevis models [9,17], respectively. The GNSS observations in China are obtained from the Crustal Movement Observation Network of China (CMONOC) during the period 2011–2017. The T and P values at the GNSS stations are interpolated in the horizontal and vertical directions using the ERA5 data. In addition, the key parameter *T_m_* is also calculated on the basis of the improved global pressure and temperature 2 wet (IGPT2w) model [18]. The IGPT2w model achieves a superior performance relative to other models in China, especially in Western China, by eliminating the major systematic errors of the GPT2W model [18]. In this work, hourly GNSS-derived PWV in China is generated during the period 2011–2017 and compared with the corresponding AERONET, radiosonde data, and ECMWF ERA-Interim products, respectively. The hourly PWV is then used to analyze the diurnal variations of atmospheric water vapor. The results of this work are interesting and significant for further regional climate studies, such as the influence of ENSO event on atmospheric water vapor in China.

## 2. Data and Method

### 2.1. Data Description

**Figure 1:**
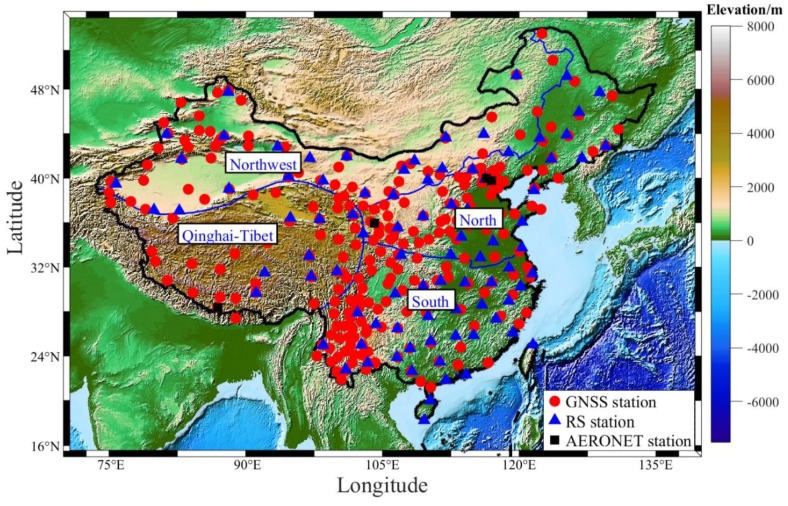
Geographic distributions of Global Navigation Satellite System (GNSS), Radiosonde (RS), and AERONET stations over mainland China. The blue line refers to the boundary line dividing China into four regions.

GNSS observations are derived from CMONOC and processed using the GAMIT/GLOBK (version 10.4) software [19]. CMONOC was built from 1997 to 2000 and operated in 2011. CMONOC includes a large number of SLR, VLBI, gravity, and GNSS stations, each of which consists of 260 reference stations and about 2,000 discontinuous stations [20]. The main purpose of CMONOC is to monitor crustal movements, gravity field shapes, and changes in the mainland of China; the variations in the water vapor content of the troposphere; and ionospheric ion concentrations. CMONOC includes two phases. The first phase covers the period from 1999 to the middle of 2010. Around 28 permanent GNSS stations were established in the Chinese region in this phase. The second phase covers the period from the end of 2010 to the present; by 2015, the number of GNSS stations had increased to about 260 [21]. Those stations exhibit high accuracy and good stability. The tropospheric product provided by CMONOC is ZTD with a temporal resolution of 1 h. As a result of the lack of ZTD time series at some stations, only 249 of the 260 GNSS stations are selected during the period 2014–2017, in this study. The corresponding GNSS stations used in the current work are presented in Figure 1.

ERA-Interim and ERA5 are the fourth- and fifth-generation reanalysis datasets of the ECMWF that cover the reanalysis data from 1979 to two to three months before the present. Both datasets provide meteorological parameters, such as P, T, and PWV, with different temporal–spatial resolutions. The temporal resolution of the ERA-Interim dataset is 6 h, and that of the ERA5 dataset is 1 h. Therefore, the ERA5 dataset is expected to be widely used in the future and gradually replace the ERA-Interim dataset. Compared with the previous generations of data products, the ERA5 reanalysis dataset was generated by using the 4D-Var data assimilation in IFS Cycle 41R2 (CY41R2) of the ECMWF Integrated Prediction System. The parameters P and T are derived from the ERA5 dataset and interpolated into the GNSS station and height with a temporal resolution of 1 h (https://www.ecmwf.int/).

Radiosonde data are also selected in the current work to validate the accuracy of P and T derived from ERA5 and PWV using GNSS and ERA5 data. The radiosonde data are derived from the Integrated Global Radiosonde Archive Version 2 (IGRA2) dataset. IGRA2 was generated in 2016 by the National Climate Data Center and contains more radiosonde stations than the previous dataset (IGRA1). This dataset includes P, T, relative humidity, and other parameters with temporal resolutions of twice or four times daily (ftp://ftp.ncdc.noaa.gov/pub/data/igra/). In this experiment, 87 radiosonde stations in China are selected (Figure 1), and 52 of them are collocated with the corresponding GNSS stations.

The PWV derived from AErosol RObotic NETwork (AERONET) with a temporal resolution of 1 h is also used to validate the generated PWV dataset at four stations during the period 2014–2017. AERONET, which is composed of a CE-318 solar photometer, mainly studies the optical and microphysical properties of global aerosols. The CE-318 solar photometer makes direct spectral measurements of solar radiation in channels between 340 and 1640 nm and in a water vapor band (936 nm). Multichannel measurements are used to retrieve aerosol optical thickness, and measurements at 936 nm are used to calculate PWV [21,22]. In the past 20 years, solar photometer-based PWV has been applied to many studies due to its high reliability and accuracy [23,24]. The three levels of PWV data provided by AERONET are raw data (Level 1.0), cloud-screened data (Level 1.5), and cloud-screened and quality-assured data (Level 2.0), respectively [25]. The corresponding PWV dataset can be obtained from the AERONET website (https://aeronet.gsfc.nasa.gov/). In this study, the PWV values derived from the Level 2.0 products are selected at the chosen four stations (Figure 1). And we arranged the characteristics of the datasets selected for the whole experiment in Table 1. Here, China is divided into four regions according to the characteristics of geographical location, natural geography, and human geography.

### 2.2. Interpolation Method

As mentioned previously, the grid-based P and T data derived from ERA5 must be interpolated into the RS stations prior to comparison; the interpolation can be completed on the basis of the following formula [26]:

(1) $$P=P_{0}{\left(1-0.0000226(h-h_{0})\right)}^{5.225}$$

(2) $$dT/dh=-0.0065{\;}^{{}^{\circ}}C/m$$

where $P_{0}$ refers to the pressure at the grid point and h and $h_{0}$ are the heights at the station and grid point, respectively. dT and dh are the differences of the T and height between station and grid point, respectively. The height system should be unified due to the different height systems used for the ECMWF, RS, and GNSS data. The data provided by ECMWF and IGRA adopt geopotential height systems, whereas the GNSS data adopt the ellipsoidal height system. In this work, the normal height system is used. The specific steps to convert normal height from geopotential height to quasi-geoid height were presented in our previous study [27].

An empirical correction formula of PWV is also used herein to unify the PWV values of the collocated stations at different heights

(3) $$PWV_{h1}=PWV_{h2}\cdot \exp(-(h1-h2)/2000)$$

where $PWV_{h1}$ and $PWV_{h2}$ are the PWV values corresponding to the heights of h1 and h2, respectively.

### 2.3. Retrieval of PWV

In this work, ZTD is derived from CMONOC. ZHD at GNSS stations can be calculated using the interpolated P on the basis of Equation (3) [9]. Therefore, the ZWD used to calculate PWV can be obtained by extracting ZHD from ZTD. The ERA5-derived T at the GNSS stations are used to calculate the *T_m_* value on the basis of the IGPT2w model proposed by Huang et al. (2019a) [14]. PWV is then obtained with Equation (4):

(4) $$ZHD=\frac{0.002277\cdot P}{1-0.00266\cdot \cos(2\varphi )-0.00028\cdot H}$$

where φ and H are the latitude (rad) and orthometric height (km) of a GNSS station, respectively. ZHD is mainly affected by P, and 1 hPa of error in P leads to a 0.2 mm error in PWV [28].

(5) $$PWV=\frac{10^{6}}{\left(K_{2}^{\prime }+K_{3}/T_{m}\right)\cdot R_{V}\cdot \rho }\cdot ZWD$$

where $K_{2}^{\prime }$, $K_{3}$, and $R_{V}$ are constants with values of 16.48 $K\cdot hPa^{-1}$, (3.776 ± 0.014) × 10^5^ $K^{2}\cdot hPa^{-1}$, and 461 $J\cdot kg^{-1}\cdot K^{-1}$, respectively. ρ is the water vapor density [29].

## 3. Evaluation of ERA5-Derived P and T over China

### 3.1. Comparison between ERA5 and ERA-Interim Products

**Figure 2:**
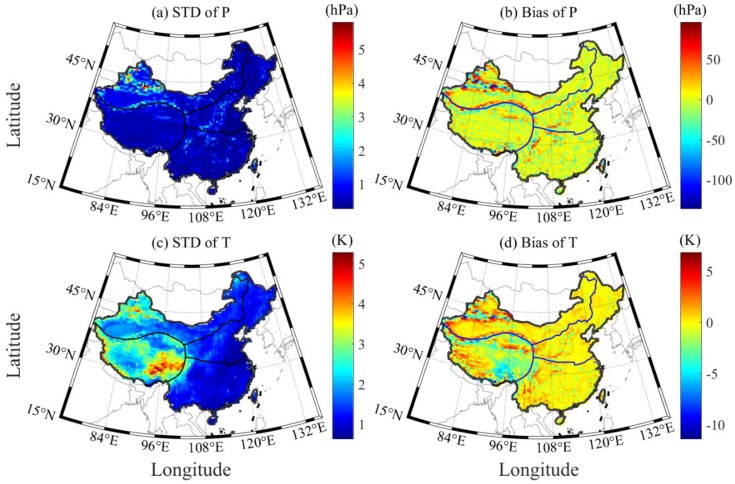
Standard Deviation (STD) and bias distributions of P and T derived from ERA5 and ERA-Interim at grid points over the period of 2005–2016 in China.

The ERA5-derived P and T products at grid points with temporal–spatial resolutions of 1 h and 0.5° × 0.5°, respectively, are first compared with those derived from ERA-Interim over China in the period of 2005–2017. China covers a vast territory with different characteristics in the east, west, north, and south regions. Hence, the country is divided into four geographical regions, namely, the northern, southern, northwest, and Qinghai Tibet regions, according to geographical location and the natural and human geographical characteristics of different regions (Figure 1). Figure 2 presents the standard error (STD) and bias of P and T between ERA5 and ERA-Interim in China during the period 2005–2007. The accuracies of P and T derived from those two types of re-analysis data are similar, but the differences of P and T are relatively large in the northwest and Qinghai Tibet regions. This deviation may be related to the complex terrain and the lack of adequate ground-based meteorological observations. The statistical results in Table 2 reveal that the average STD and bias of P and T in China are 0.71/−0.36 hPa and 1.77/−0.33 K, respectively. Therefore, it can be concluded that the P and T data of ERA5 and ERA-Interim in most regions of China have good consistency. Because the time resolution of ERA5 data is higher than the ERA-interim data, the ERA5 data set is likely to replace ERA-interim data and widely used in the future.

### 3.2. Comparison of ERA5 Products with Radiosonde Data

**Figure 3:**
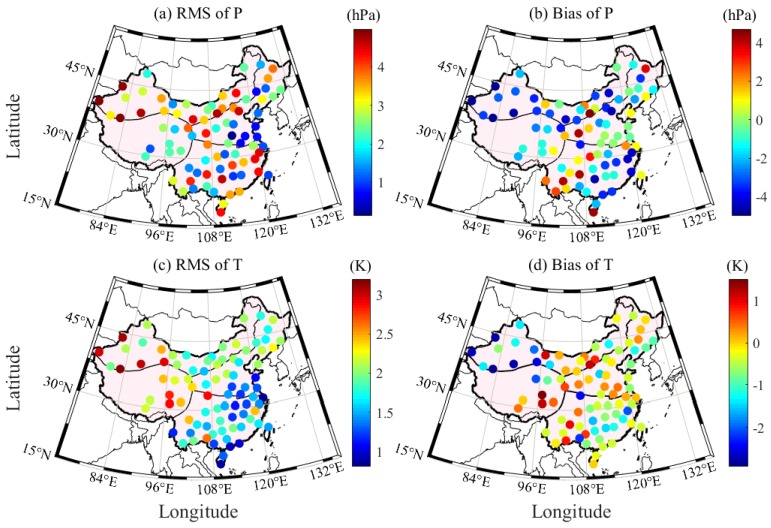
RMS and bias of P and T derived from ERA5 at 87 RS stations during the period 2005–2017 in China.

The P and T derived from ERA5 are used to interpolate the corresponding values at the RS station and height during the period 2005–2017 in China. The interpolated P and T at the RS stations are validated with the RS-derived P and T, respectively. In the experiment, the differences of the ERA5-/RS-derived P and T larger than 3σ are regarded as gross errors and thus removed. The accuracies of P and T derived from ERA5 can subsequently be calculated. Figure 3 presents the root mean square (RMS) and bias of P and T derived from ERA5 at 87 RS stations during the period 2005–2017 in China. Table 3 provides the specific statistical results of a number of RS stations and the average RMS and bias of the ERA5-derived P and T in the four regions of China. It can be found that the ERA5-derived P and T in northern and southern China are of good quality, whereas the accuracies of the values in the northwest and Qinghai Tibet regions are slightly poor. The statistical results reveal that the average RMS and bias of P and T in China are 2.71/−1.11 hPa and 1.88/−0.51 K, respectively. Such results are consistent with those in Section 3.1 and are thus enough to verify the good performance of the ERA5-derived P and T in China.

## 4. Hourly PWV Derived from CMONOC and ERA5

### 4.1. Analysis of GNSS-Derived ZTD from CMONOC

**Figure 4:**
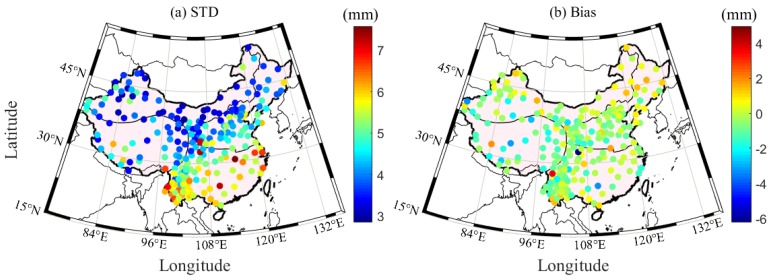
STD and bias distributions of GNSS-derived ZTD differences using GAMIT/GLOBK and PANDA software during the period 2011–2017.

To validate the consistency of the GNSS-derived ZTD obtained on the basis of GAMIT/GLOBK (version 10.4) software with that processed by other softwares, the corresponding ZTD parameters calculated with the PPP technique using the positioning and navigation data analyst (PANDA) package are applied [30,31]. PANDA software has been independently developed by the research center of satellite navigation and positioning technology of Wuhan University since 2001, and has been adopted by many famous international research institutions. Its goal is to realize the post-processing and real-time processing analysis of GNSS/SLR/DORIS/VLBI and other kinds of observation data [32].The double difference mode is used in the GAMMIT/GLOBK software while the non-difference mode is applied in the PANDA package. A total of 246 out of 260 GNSS stations are selected during the period 2011–2017 in China. The ZTD differences larger than 3σ are removed as gross errors. Figure 4 presents the STD and bias distributions of the GNSS-derived ZTD differences using the GAMIT/GLOBK and PANDA software during the period 2011–2017. Table 4 provides the statistical results of the STD and bias, as well as the number of RS stations, in four regions of China. As shown in Figure 4, the STD and bias in mainland China are considerably small, and the statistical results reveal that their values are 4.6 and −0.4 mm, respectively. Table 4 indicates that the ZTD error is slightly large in southern China with an RMS of 5.5 mm; this region is mainly affected by the large atmospheric water vapor content. The RMS value is approximately 3.7 mm in northwest China, where the atmospheric water vapor is low.

### 4.2. Theoretical Error of PWV Calculated Using the Hourly P and T from ERA5

The theoretical error of PWV can be deduced using the error propagation law according to the accuracies of P, ZTD, and *T_m_*. In our experiment, the accuracies of P, ZTD, and T are approximately 2.7 hPa, 5 mm, and 1.9 K, respectively. Therefore, the theoretical error of PWV in this work can be calculated on the basis of the following formula [33]:

(6) $$\begin{array}{l} \sigma_{v}=\sqrt{{\left(\frac{\sigma_{ZTD}}{Q}\right)}^{2}+{\left(\frac{2.2767\sigma_{P_{0}}}{f(\lambda ,H)Q}\right)}^{2}+{\left(\frac{P_{0}\sigma_{c}}{f(\lambda ,H)Q}\right)}^{2}+\left(V\frac{\sigma_{Q}}{Q}\right)}\\ f(\lambda ,H)=(1-2.66\times 10^{-3}\cos(2\varphi )-2.8\times 10^{-7}H)\\ \sigma_{Q}=10^{-6}\rho_{w}R_{v}\sqrt{{\left(\frac{\sigma_{k_{3}}}{T_{m}}\right)}^{2}+\sigma_{k_{2}^{\prime }}^{2}+{\left(k_{3}\frac{\sigma_{T_{m}}}{T_{m}^{2}}\right)}^{2}} \end{array}$$

where $\sigma_{v}$, $\sigma_{ZTD}$, $\sigma_{P_{0}}$, and $\sigma_{Q}$ represent the errors in the ZTD, P, T, and conversion factor, respectively. V refers to PWV, and $\sigma_{C}=0.0024$, Q is a parameter for converting ZWD to PWV with an average value of 0.16. f(λ,H) accounts for the variation of gravitational acceleration at the ellipsoidal latitude φ and ellipsoidal height H of the GNSS station; it is generally considered to be equal to 1. In addition, $\sigma_{Q}$ is the effect of *T_m_*, which can be neglected for most cases.

Therefore, the final theoretical error of PWV can be calculated using Equation (6) based on the obtained errors in P, T, ZTD and conversion factor. Table 5 shows the statistical mean values of the four regions. It can be found that the average theoretical error of PWV in the whole of China is about 1.85 mm. The maximum value occurs in the Qinghai-Tibet area, with the value being approximately 2 mm; whereas the minimum value is noted in northern China, with the value being about 1.3 mm.

### 4.3. T_m_ Calculation Using Improved GPT2w (IGPT2w) Model

**Figure 5:**
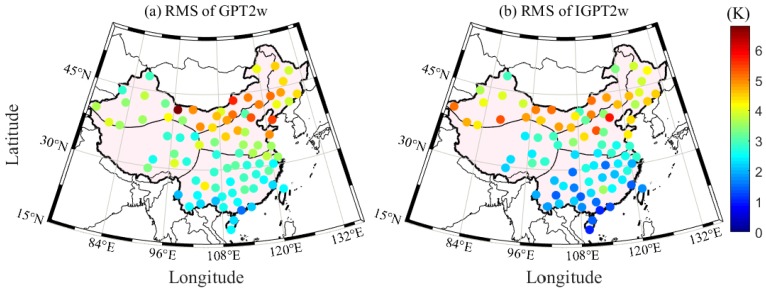
RMS distributions of *T_m_* differences derived from GPT2w and IGPT2w at 87 radiosonde stations when the RS-derived *T_m_* is considered as the reference.

**Figure 6:**
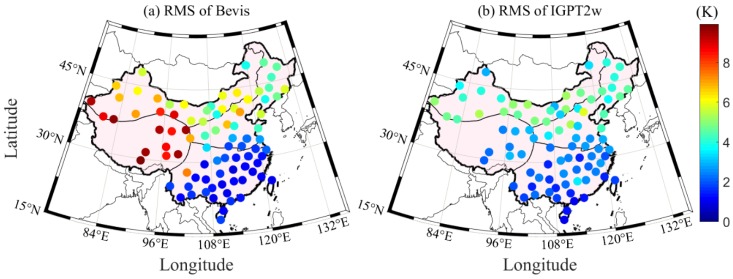
RMS distributions of *T_m_* differences derived from Bevis and IGPT2w at 87 radiosonde stations when the RS-derived *T_m_* is considered as the reference.

As described in Equation (2), *T_m_* is the only parameter affecting the accuracy of PWV when ZWD is known. Therefore, how to obtain a high precision *T_m_* using the interpolated T at a GNSS station is the key point in this section. The GPT2w model is one of the most widely used models for calculating *T_m_*, but it is prone to evident systematic bias [34]. The main reason is that the vertical correction of *T_m_* is not considered in the GPT2w model. To overcome this issue, Huang et al. (2019) proposed an IGPT2w model, which considers the height correction of *T_m_*; such a model has greatly corrected the systematic error in the western region of China relative to the GPT2w model [18]. The corresponding vertical correction of *T_m_* is presented in Equation (7). Therefore, the IGPT2w model is used in this work to calculate the *T_m_* value of each GNSS station. Figure 5 presents the RMS distributions of the *T_m_* differences derived from the GPT2w and IGPT2w models at 87 radiosonde stations when the RS-derived *T_m_* is considered as the reference. The accuracy of the IGPT2w-derived *T_m_* in China, especially the southern region, shows an improvement. The statistical result reveals that the average improvement rate of *T_m_* using the IGPT2w model is approximately 10% in China. Figure 6 gives the comparison experiment of *T_m_* calculated by Bevis formula and IGPT2w model. It can be seen from the figure that the accuracy of *T_m_* derived from IGPT2w is higher than that from Bevis formula, so we chose the IGPT2w model to calculate *T_m_* in this paper.

(7) $$\begin{array}{l} T_{m}^{t}=T_{m}^{r}+\gamma \times (\delta h_{t}-\delta h_{r})\\ T_{m}^{r}=\beta_{0}+\beta_{1}\cos\left(2\pi \frac{doy}{365.25}\right)+\beta_{2}\sin\left(2\pi \frac{doy}{365.25}\right)+\beta_{3}\cos\left(4\pi \frac{doy}{365.25}\right)+\beta_{4}\sin\left(4\pi \frac{doy}{365.25}\right)\\ \gamma =\alpha_{0}+\alpha_{1}\cos\left(2\pi \frac{doy}{365.25}\right)+\alpha_{2}\sin\left(2\pi \frac{doy}{365.25}\right)+\alpha_{3}\cos\left(4\pi \frac{doy}{365.25}\right)+\alpha_{4}\sin\left(4\pi \frac{doy}{365.25}\right) \end{array}$$

where $T_{m}^{t}$ is the $T_{m}$ calculated by the GPT2w model at the target height while $T_{m}^{r}$ is the $T_{m}$ calculated at the height of the grid point. $\delta h_{t}$ and $\delta h_{r}$ are the heights at the target and grid point in km, respectively. doy is day of year. $\alpha_{0}$ represents the mean value of the decreasing rate of $T_{m}$. $\left(\alpha_{1},\alpha_{2}\right)$ and $\left(\alpha_{3},\alpha_{4}\right)$ are the coefficients of the annual and semiannual periods, respectively. $T_{m}^{t}$ is the $T_{m}$ calculated by the GPT2w model at the target height.

## 5. Validation and Analysis of Hourly PWV Dataset

### 5.1. Comparison of Hourly PWV Dataset with AERONET Data

**Figure 7:**
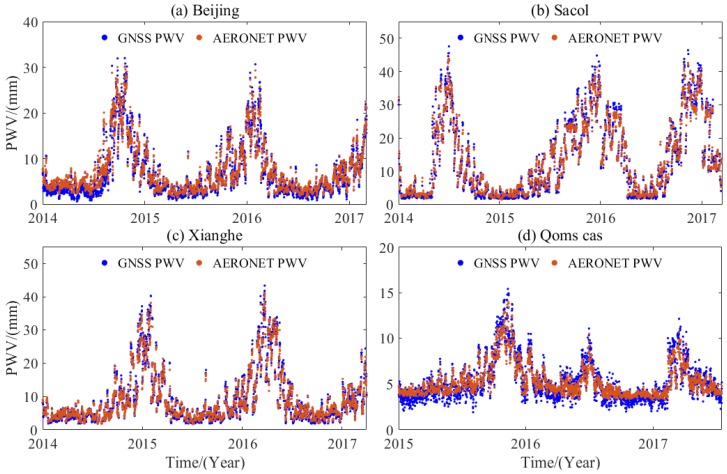
Time series of hourly PWV derived from AERONET and GNSS at four collocated stations during the period 2014–2017.

After the *T_m_* is calculated, the hourly PWV dataset at each GNSS station can be obtained on the basis of the GNSS-derived ZTD and ERA5 data during the period 2011–2017. To validate the hourly PWV dataset, this study selects and compares four collocated stations between AERONET and GNSS. Figure 7 presents the time series of the hourly PWV derived from AERONET and GNSS at the four collocated stations. It can be observed from Figure 7 that the GNSS-derived PWV agrees well with that from AERONET at the four collocated stations. The calculated correlation coefficients between the GNSS- and AERONET-derived PWV are 0.97, 0.99, 0.99, and 0.93. A high correlation indicates that the generated hourly PWV dataset is of good quality. The statistical results reveal that the RMS values of the GNSS-derived PWV at the Beijing, Sacol, Xianghe, and Qoms_cas stations are 1.13, 2.04, 2.04, and 1.41 mm, respectively.

### 5.2. Comparison of Hourly PWV Dataset with RS Data

**Figure 8:**
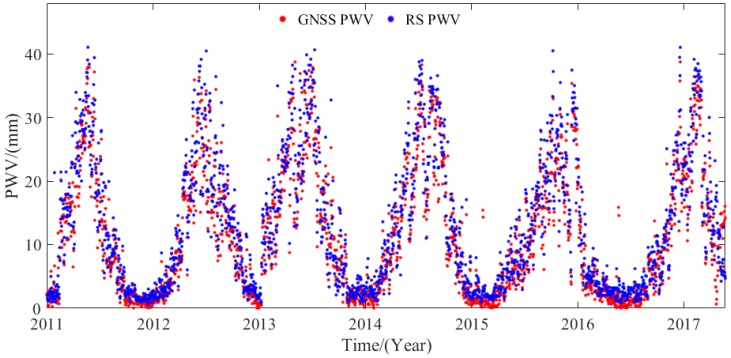
Time series of PWV derived from GNSS and RS at HLAR station (49.3° N, 119.7° E) during the period 2011–2017.

**Figure 9:**
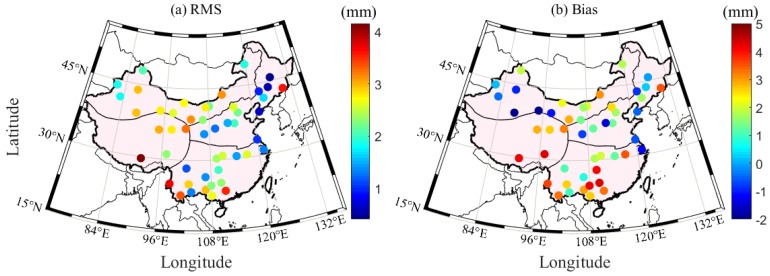
RMS and bias distributions of PWV differences derived from GNSS and RS during the period 2011–2017.

To further evaluate the accuracy of the hourly PWV dataset in China, this study selects the GNSS and radiosonde data from 52 out of the 87 collocated stations in China during the period 2011–2017. Herein, the horizontal distance less than 60 km in the latitudinal and longitudinal directions and the vertical difference between the GNSS and radiosonde stations less than 500 m are used as a principle to judge whether the radiosonde and GNSS stations are collocated or not. The empirical formula in Equation (3) is used to unify the PWV value from radiosonde height to GNSS height. Figure 8 presents the time series of the GNSS- and RS-derived PWV at the HLAR station (49.3° N, 119.7° E) during the period 2011–2017. A good consistency exists between the GNSS- and RS-derived PWV. Figure 9 presents the RMS and bias distributions of the PWV differences derived from GNSS and RS at 52 stations during the period 2011–2017. The statistical result reveals that the average RMS and bias are 2.25 and 1.57 mm, respectively. Table 6 provides the specific RMS and bias in the four regions of China. Excluding that of the Qinghai-Tibet region, the RMS values in the other three areas are less than 3 mm. Such a result obtained above indicates the potential application of the hourly PWV dataset generated in this work in weather nowcasting [35]. The average RMS is larger than the theoretical value, and this phenomenon is acceptable because of the uncertainties in radiosonde PWV data and the error introduced when using the empirical formula in Equation (3) [11].

### 5.3. Comparison of PWV Image with ERA5

**Figure 10:**
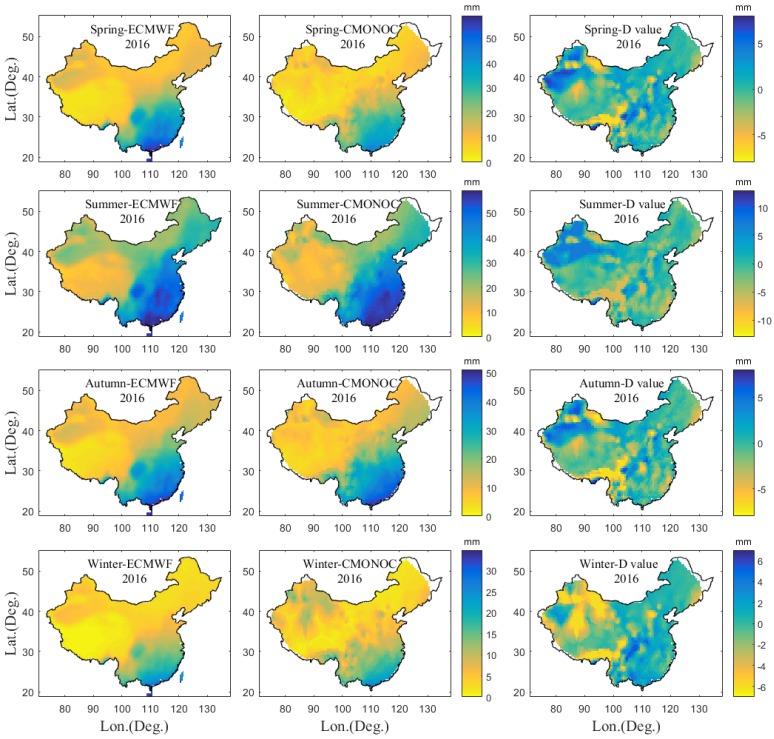
Mean PWV image derived from GNSS and ERA5 in different seasons and their difference distributions in 2016.

Apart from the comparison with the GNSS-derived PWV at collocated stations, the PWV image is also compared with that from the ERA5. A total of 246 GNSS stations are used to construct the Delaunay triangulation net in China, and the hourly PWVs of three points of a Delaunay triangle are used to interpolate the PWV value at the grid point with the spatial resolution of 0.5° × 0.5°. Figure 10 provides the mean PWV image derived from GNSS and ERA5 in different seasons in 2016 and their difference distribution. It can be observed that the PWV distribution derived from GNSS agrees well with that from ERA5 under different seasons, but the PWV difference is slightly larger in summer. The statistical results reveal that 91%, 81%, 90%, and 87% of the areas in China have PWV differences of less than 5 mm in four seasons, respectively.

### 5.4. Analysis of Diurnal PWV Variations in China

**Figure 11:**
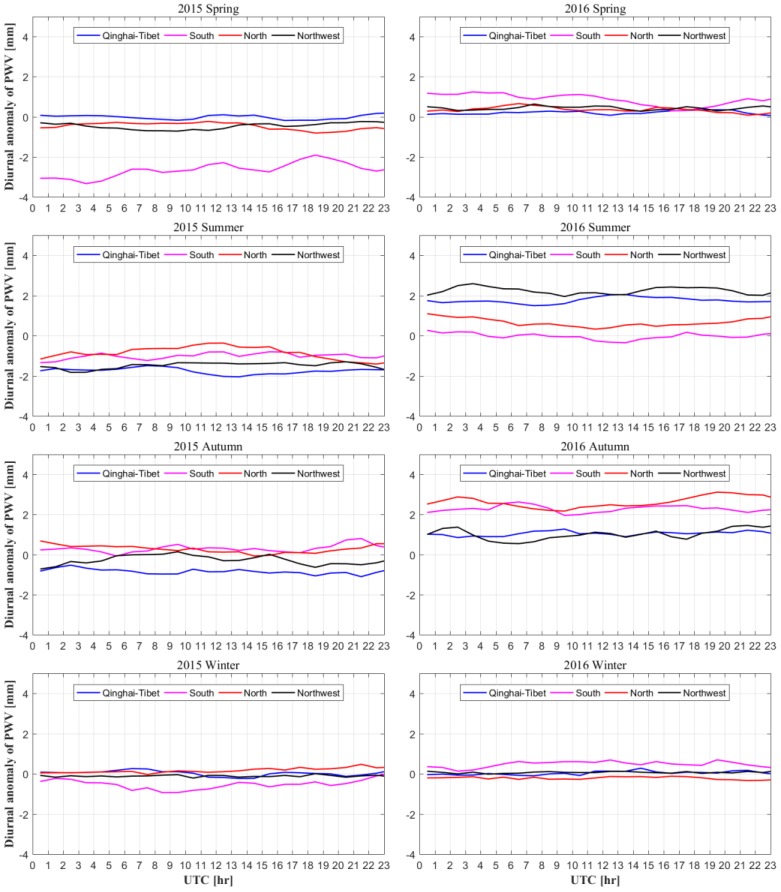
Hourly diurnal anomalies of PWV time series in the four regions of China in different seasons during the period 2015–2016.

Hourly PWV is generated in this work to calculate the diurnal variations of atmospheric water vapor, which is an important indicator for climate monitoring. Therefore, the diurnal anomalies of PWV in the four regions of China for four seasons are calculated. The hourly diurnal anomaly of PWV is calculated by removing the average PWV of the corresponding season. Spring, summer, autumn, and winter are from March to May, June to August, September to November, and December to February, respectively. Figure 11 presents the hourly diurnal anomalies of the PWV time series in the four regions of China under different seasons during the period 2015–2016. It can be found that the diurnal anomalies of the PWV in four seasons in 2016 were below and around 0 mm and increased to above 0 mm to a different extent gradually. In terms of season, this phenomenon is especially obvious in summer and autumn. Regionally speaking, the phenomenon is most obvious in the southern region. The main reason is the El Niño/Southern Oscillation (ENSO) event in 2016–2017. Heavy rainfall occurred in southern China, especially in summer and autumn. This condition led to a decrease in atmospheric water vapor content in the troposphere. Therefore, the hourly diurnal anomaly of PWV shows a decreasing trend with values below and around 0 mm. In 2017, the atmospheric water vapor returned to its normal average level, as reflected by the increasing diurnal anomaly of PWV.

## 6. Conclusions

An hourly PWV dataset is generated at 246 GNSS stations during the period 2011–2017 in China using the GNSS observations from CMONOC. The meteorological parameters at the GNSS station and height are interpolated at the horizontal and vertical directions using the ERA5-derived hourly P and T data with temporal–spatial resolutions of 0.5° × 0.5° and 1 h, respectively. China is divided into four regions, and the quality of the ERA5-derived P and T is evaluated using the ERA-Interim and radiosonde data during the period 2006–2017. The comparison with 84 radiosonde stations reveals that the average RMS and bias of P/T in China are 2.71/−1.11 hPa and 1.88/−0.51 K, respectively. GNSS-derived ZTDs at 246 stations from CMONOC are analyzed on the basis of two processing modes, and the STD and bias of ZTD in China are about 4.7 and −0.47 mm, respectively. Therefore, the theoretical error of PWV caused by errors in ZTD, P, and T is approximately 1.9 mm according to the law of error propagation. A practical validation of the hourly PWV dataset at 264 GNSS stations is performed, and the results are compared with the AERONET-/RS-derived PWV data at collocated stations. The outcomes show that the average RMS and bias of the hourly PWV dataset are 2.25 and 1.57 mm, respectively. In addition, the PWV image is compared with that from ERA-Interim under different seasons in 2016. The comparison highlights the good performance of the generated PWV dataset in the whole of China. The diurnal variation of the PWV time series is also analyzed under different seasons in 2016 and 2017, and the result indicates that the hourly PWV dataset is capable of reflecting the response of atmospheric water vapor to climate events, such as the ENSO event.

## Figures and Tables

**Table 1:** Characteristics of datasets selected in the experiment.

Data	Spatial and Temporal Resolution		Temporal Coverage/Year	Sources
ECMWF-Derived P and T	0.5° × 0.5°	hourly	2005–5017	https://www.ecmwf.int
CMONOC-Derived ZTD	station	hourly	2011–2017	ftp://ftp.cgps.ac.cn/
RS-Derived P, T and PWV	station	12 h	1957–2016	ftp://ftp.ncdc.noaa.gov
AERONET-Derived PWV	station	hourly	2001–2017	http://aeronet.gsfc.nasa.gov

**Table 2:** Statistical results of Standard Deviation (STD) and bias of P /hPa and T/K in four regions of China.

Area	P/hPa		T/K	
	Bias	STD	Bias	STD
South	−0.53	0.52	−0.15	1.13
Qinghai-Tibet	−2.54	0.59	−1.24	2.71
northwest	1.95	1.05	0.09	1.87
north	−0.47	0.58	−0.09	1.25
china	−0.36	0.71	−0.33	1.77

**Table 3:** Statistical results of a number of RS stations used and the average RMS and bias of ERA5-derived P and T in four regions of China during the period 2005–2017.

Area	Total Station	P/hPa			T/K		
		Station Utilization (%)	RMS	Bias	Station Utilization (%)	RMS	Bias
North	20	100	2.16	−0.54	100	1.74	−0.30
South	34	88	2.45	−0.70	100	1.66	−0.59
Northwest	24	100	2.66	−2.12	100	2.20	−0.87
Qinghai-Tibet	9	100	2.90	−1.24	100	2.40	0.10
China	87	95	2.71	−1.11	100	1.88	−0.51

**Table 4:** Statistical results of STD and bias, as well as the number of RS stations, in the four regions of China during the period 2011–2017.

Area	Total Station	Station Utilization (%)	STD (mm)	Bias (mm)
North	69	100	4.37	−0.24
Qinghai-Tibet	38	97	4.50	−0.80
South	85	96	5.50	−0.42
Northwest	52	100	3.69	−0.32
China	244	98	4.60	−0.40

**Table 5:** Theoretical error of precipitable water vapor (PWV) calculated on the basis of the error propagation law in China.

Area	Theoretical Error of PWV (mm)
North	1.29
South	1.35
Northwest	1.54
Qinghai-Tibet	1.97
China	1.85

**Table 6:** Statistical results of RMS and bias in four regions of China over the period of 2011–2017.

Area	Total Station	Station Utilization (%)	RMS (mm)	Bias (mm)
North	13	100	1.53	0.61
Qinghai-Tibet	7	71	3.09	3.44
South	18	94	2.35	2.43
Northwest	14	100	2.52	0.73
China	52	94	2.25	1.57
